# Prevalence of Adjustment Problem and Its Predictors among First-Year Undergraduate Students in Ethiopian University: A Cross-Sectional Institution Based Study

**DOI:** 10.1155/2018/5919743

**Published:** 2018-09-25

**Authors:** Getu Belay Ababu, Asmare Belete Yigzaw, Yihunbelay Dinku Besene, Wondale Getinet Alemu

**Affiliations:** ^1^Department of Psychiatry, College of Medicine and Health Sciences, Dilla University, Dilla, Ethiopia; ^2^Amanuel mental specialized Hospital, Addis Ababa, Ethiopia; ^3^Department of Psychiatry College of medicine and health science, University of Gondar, Gondar, Ethiopia

## Abstract

**Background:**

Being in a university for the first time is an unpleasant event and time of heavy pressure relating to social things and dissatisfaction for several new faculty students. Adjustment difficulties are the foremost common issues among freshman students; World Health Organization area unit researched a full-of-life adjustment innovation in universities. Despite this reality, there is a few literature that identifies prevalence and factors related to the adjustment downside among university/college students.

**Methods:**

Institutional based quantitative cross-sectional study style was conducted from May to June 2016. A total of 537 haphazardly elite students concerned within the study. The result adjustment downside was explained with Student Adjustment to College Questionnaire (SACQ). Data was described using descriptive analysis; logistic regression was used to assess the relation between adjustment downside and associated factors. An odds quantitative relation 95% confidence interval was used to point out the strength of association. Applied mathematics significance was declared as a p value less than 0.05.

**Result:**

A total of 537 respondents participate with a response rate of eighty-five percent. the prevalence of adjustment downside among the respondents was 228 ( 42.5%). Of the respondents, 327 (60.9%) were male and therefore the mean (sd) age was 19 (+- 1) years. Being away from home and homesickness (AOR=4.15,95% CI: 2.46,2,99 and AOR=5. 9,95% CI: 2.35,14.76) and difficulties in socializing or making friends (AOR=3.99,95% CI:2.29,6.98) and difficulties in managing time and study skill (AOR=3.02,95% CI: 1.3,7.02) were abundant associated factors with adjustment downside.

**Conclusion:**

The study confirms that freshman students joining university do face problems in adjusting themselves at the university. Homesickness, difficulties in socializing or making friends, and difficulties in managing time and study skill were found to be significant factors in adjustment downside.

## 1. Background

University students are always exposed to psychosocial stressors throughout teaching years of a personal square showing emotionally and intellectually a lot of sternness nearly compared to the other stage of education. At this stage, students face loads of pressures and challenges that create a spread of physical, social, and emotional difficulties [1]. The majority of first-year students have stated that transition to campus happens to be the most stressful adjustment phase in their lives [2].

Adjustment is the psychological processes accustomed to adapt, cope, and manage the issues sweet-faced in the standard of living. For university students, the adjustment could be a multifaceted side that may be divided into four completely different elements that square measure educational, social, Personal-emotional adjustment and attachment to the university [3].

According to DSM-IV-TR adjustment disorder outlined due to the development of emotional or activity symptoms in response to classifiable stressor(s) occurring at intervals three months of the onset of the stressor(s) [4], these symptoms or behaviors are clinically important as proved by either of the following: marked distress that is far more than what would be expected from exposure to the agent and important impairment in social or activity (academic) functioning [3, 5].

Adjustment difficulties arise from the variations between the expectations of the scholars and realities of field life [6].

Studies conducted on young people indicate that those who live with their parents are less likely to have emotional issues and their behavior is more likely to be under their parents' management [7]. Adjustment disorder has calculable incidence of 5–21% in psychiatry consultation services for adults [8]. Forty-eighth of freshman students in Jimma University experience a high level of total adjustment issues [9].

Multiple factors are considered as risk factors for adjustment problems like coping strategy, social support, the new values, norms and surprising standards that are overwhelming, educational demands, financial problems and homesickness, social stressors related to communication barriers, being female, having low socioeconomic standing, disorientation and culture shock, and different educational expectations [9–11].

Entering college is taken into account as a time of full of stress; several emotional and psychological problems are developed as a result. Since these students place the majority of their time, energy, and cash into it [12–14].

Findings within the space of student adjustment method and difficulties indicate that if students are not able to normalize their state of mind to the potential challenges they might face in universities, there is bigger probability of refraining from their studies and it normally ends up in suicide [15–17].

There is a restricted analysis on assessing the adjustment processes of scholars in universities and not a lot of work is done on student adjustment and connected interventions in African nation [18]. It is crucial to convey correct attention to the issues involving adjustment among freshmen students [6].

Despite all these problems, there is a scarcity of revealed studies in African nation. Thus, the aim of this study was to assess the prevalence of adjustment disorder and associated factors among university students.

## 2. Methods

### 2.1. Study Design

Institutional based cross-sectional study design was used.

### 2.2. Study Area and Period

The study was conducted in Dilla University from May to June 2016, located in south of the capital city of Ethiopia and far by 360 Km. One of the newer universities that was founded in 1996 G.C is Teachers and Health Science College in Ethiopia. However, since 2007 G.C, it is providing higher level of education in many disciplines, which has been clustered into three campuses and six colleges. Currently, it has 47 undergraduate and 17 postgraduate departments, at BA/ BSc, Bed, MA/MSc level with regular, extension, and summer courses, and it has about 30108 students.

### 2.3. Study Population

The participants of this study were all first-year regular undergraduate students of Dilla University during the study period. Single population proportion formula (with the assumption of 5% margin of error, 95% confidence level and 50% proportion) was used to calculate sample size; and it was found to be 423 (including 10% nonresponse rate), to reduce bias effect multiplied by 1.5; then it was 633.

After campuses were stratified and sample size proportionally allocated, the required sample size of study subjects or students was selected by simple random sampling from each college/school/department. The study was initially approved by the ethical review board of Dilla University. Written informed consent was sought for each participant who volunteered and fulfilled the inclusion criteria. Participants with age greater than or equal to 18 years were included and those who were unable to speak or hear were excluded from the study.

### 2.4. Data Collection Procedures

Data was collected using structured self-administered questionnaire having three parts: first, sociodemographic characteristics and, second, Student Adaptation to College Questionnaire (SACQ), developed by Baker and Siryk, 1999, and standardized and published by Western Psychological Service (WPS) consisting of 67 self-rating responses used to assess students' adjustment to college in light of past researches and surveys. Using a five- (5-) point scale ranging from one to five representing “strongly agree” to “strongly disagree” was used as primary data collection instrument. It has scored from 67 to 335 and cutoff point <=201 score for adjustment problem. It is divided into four principal subscales that focus on four aspects or dimensions of adjustment to college: academic adjustment (24 items; =.84), social adjustment (19 items; alpha=.84), personal-emotional adjustment (14 items; alpha= .81), and institutional attachment or commitment (8 items; alpha =.80). With two additional items of general adjustment, overall adjustment has 67 items (alpha=.91) [19, 20]. The questionnaires were translated into Amharic (local language) by an Amharic speaking linguist. The back translation was performed by mental health specialist in English and then consensus version was developed in a group discussion by involving the research team. This was compared with the original version and confirmed to be satisfactory for use. The questionnaires were tested on 5% of the samples in Hawassa University to make it easier to understand.

### 2.5. Data Collection and Analysis

First, the data were checked for completeness and consistency, and then it was coded. The coded data were entered into epidemiological information (EPI info) software version 7 and exported to statistical package for social sciences (SPSS) version 20 for further statistical analysis. Descriptive analysis was done using frequency and proportion; mean, standard deviation and frequency tables and graphs were used for presenting the data. Logistic regression analysis was done to assess factors associated with adjustment problem. The multivariate logistic regression was used to control for confounding to identify the independent predictor of adjustment. The finding was determined using crude and adjusted OR with 95% confidence interval in the bivariate and multivariate analysis. Variables with P value less than 0.05 were declared to be statistically significant.

## 3. Results

A total of 537 participants were enclosed within the study, yielding a response rate of eighty-five percent. Ninety-six respondents did not fill all the questions properly, twenty-four returned back questionnaires without responding to questions, and the remaining seventy-two respondents did not return the questionnaires altogether. The mean (SD) age of the respondents was 19(±1) years. Among the respondents, 220 (41%) were from the College of Engineering and Technology.

Near half of the respondents, 235 (43.8%), were orthodox faith followers and twenty-four percent were from Oromos. relating to respondents' parental marital condition the majority of eighty-three percent oldsters were married whereas the remainder sixteen were within the alternative marital condition like unmarried, separated in conflict, and single (Table 1).

### 3.1. Adjustment Problem of the Respondents

**Figure 1:**
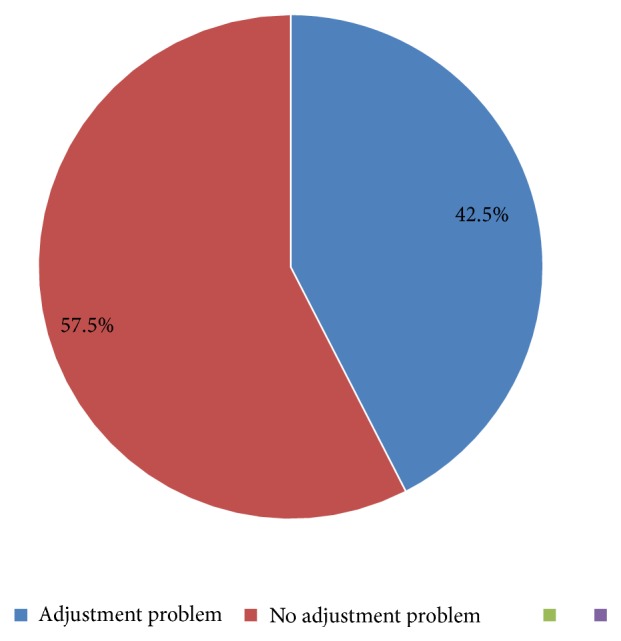
Prevalence of adjustment problem among university students in Dilla University, Southern Ethiopia, 2016 (N=537).

Two hundred twenty-eight (42.5%) respondents have overall adjustment problem (Figure 1).

**Figure 2:**
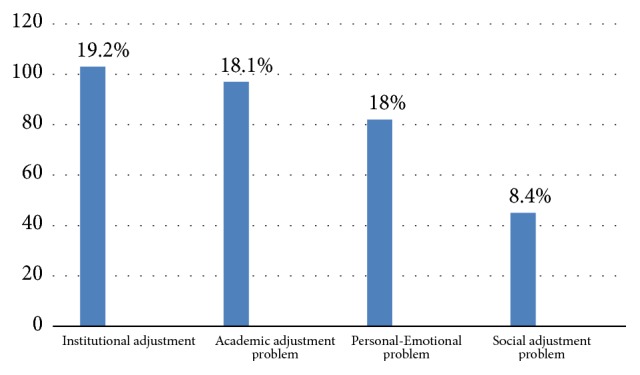
Prevalence of different adjustment problem among university students in Dilla University, Southern Ethiopia, 2016 (N=537).

Among the study subjects, a considerable variety of respondents, 103 (19.2%) and 97 (18.1%), had institutional adjustment and academic adjustment problems, respectively. On the contrary 82 (18.1) and 45 (8.4%) respondents had personal-emotional problem and social adjustment problems, respectively (Figure 2).

### 3.2. Factors Related to Adjustment Issues

Bivariate analysis was done to look at the association between adjustment problem and every determinant. Among the factors, use of psychological counseling, receiving academic assistance or study counseling, being away for the first time and homesickness, change in living arrangements or living in dormitories, difficulties with socializing or making friends, completely new and different social network and environment, difficulties in managing time and study skill, adjusting to university classes and accompanying workload, and health problem had p value less than 0.2 significance level.

Determinants that had quantity associations at p value less than 0.2 were then entered into multivariate logistic regression. In statistical method the variables with important association were being away from home, family, and friends for the first time and homesickness (AOR=4.15,95%CI:2.46,2.99 and AOR=5.89,95%CI:2.35,14.76), difficulties in socializing or making friends (AOR=3. 99,95% CI: 2.29,6.98), and managing time and study skill (AOR=3. 02,95% CI: 1.03,7.02) (Table 2).

## 4. Discussion

The aims of this cross-sectional study were to assess the prevalence of adjustment problem and associated factors among university students in Dilla University. The general prevalence of adjustment issues among freshman regular students was 42%. This study clearly indicates that adjustment problem is changing into a priority among freshman university students.

Studies done previously suggest that social and emotional adjustments are more important in making a successful transition to college than academic adjustment [21]. The most common symptom category of adjustment disorder reported by our students was institutional adjustment 19.2%. A similar number of students reported both academic adjustment problems and personal-emotional adjustment problem (18.1%) equally. A limited but still large number of students reported social adjustment problems (8.4%). Our data, similar to previous researchers who have shown that the institutional adjustment is far more than an academic adjustment, shows that social and emotional factors are extremely important in making a successful transition [22].

The general prevalence of this study was in line with the study done in Malaysia and North Jordan that was 42.8 and 50 percent of universities students having adjustment problems [2, 23]. On the other hand the prevalence of the recent study is slightly on top of the study done in Jordan University that was 36.8 % [2]. The distinction may be because of Likert scale measure; they used 9 Likert scale measurement whereas this study used 5 Likert scale.

A study conducted at Jimma University showed that the prevalence of adjustment problem was forty-eight which was slightly on top of this study finding. This might proceed to the distinction of sample size that was two hundred fourteen students concerned within the Jimma study [9].

Being away from home, family, and friends for the first time and homesickness were significantly related to the adjustment problem quite fourfold and 5 times (AOR=4. 15, 95%, CI: 2.46, 2.99 and AOR=5.90, 95% CI: 2.35, 14.76) respectively, as compared to students who had no adjustment problem. This indicated that students who have homesickness had the most common specific problems reported with association to student's adjustment problem with depressed mood being at a higher risk of developing major depressive disorder [22]. The achievable reason may be students who are away from home for the first time having difficulties to effectively study their educational work [24].

Difficulties in managing time and study ability were conjointly related to adjustment problem around four times with those who had no adjustment problem (AOR=3. 99, 95%, CI: 1.30, 7.02). This finding is supported by other study done in University of Wales, College of Cardiff; lack of time for family and friends is also important source of stress [25]. High stress levels in students may affect memory, concentration, and problem-solving ability and may compromise learning, coping, and academic performance.

The odds of participants who had difficulties of socialization and making friends were three times the odds of not having difficulties of socialization and making friends (AOR=3.02,95%, CI:1.30,7.02). The reason may be that being associated alone will increase adjustment problem [24]. This is similar to study done by international students attending United States universities and, nursing students in Rome, Italy, respectively [26, 27]. Students that believe they had a stronger social support network perceived themselves to be more capable of coping with stressful academic situations, which suggests that a promising way can ease problems of academic adjustment and vice versa. Socialization is an effective way to decrease adjustment problem and that support seeking is an important predictor of future academic performance of first-year students. Social isolation may prevent the development of communication skills and team working of students.

Furthermore, researches show that demographic characteristics like gender are related to university adjustment problem, although no significant differences are found between male and female students' adjustment level which is inconsistent with previous studies [15, 28].

### 4.1. Strength of the Study

This study is the first of its kind within the study space to point out the prevalence of adjustment disorder and associated factors among university students.

### 4.2. Limitations of the Study

This study has some necessary limitations that ought to be unbroken in mind once decoding the results. Because our study had a cross-sectional design, we could not establish causal relationships between the associations we observed. Then, longitudinal studies are necessary to establish which variables show true causal relationships with adjustment disorder in university students.

## 5. Conclusion

Near half of the study participants have adjustment problem. The prevalence of adjustment disorder was found to be high. In general, this study demonstrated increased understanding of adjustment phenomena among university students in Ethiopia and it has added to our knowledge about the factors that affect the adjustment process among university students. From this perspective, the researcher concludes that homesickness, difficulties in socialization or making friends, and difficulties in managing time and study skill were found to be a major issue for adjustment problem. Thus, authors urged that screening for adjustment problem for various kinds is ought to be suggested.

## Figures and Tables

**Table 1:** Distribution of freshman students at Dilla university, Dilla, Southern Ethiopia, 2016 (N=537).

Characteristics	N	%
Sex		
Male	327	60.9
Female	100	39.1
Age (Years)		
18-20	437	81.38
21-24	100	18.62
Marital status		
Single	521	97
Married	16	3
School		
Engineering and technology	220	41
Social and humanities	53	9.9
Business and economics	87	16.2
Educational and behavioral sciences	28	5.2
School of law	8	1.5
Natural and computational sciences	56	10.4
Health and medical science	19	3.5
Religion		
Orthodox	235	43.8
Muslim	126	23.5
Protestant	149	27.7
Others*∗*	27	5
Ethnicity		
Amhara	104	19.4
Gurage	36	6.7
Oromo	133	24.8
Sidama	45	8.4
Tigrie	62	11.5
Wolita	52	9.7
Others*∗∗*	105	19.6

*∗*Wakefeta, Catholic.

*∗∗*Siltie, Hadya, and Kembata.

**Table 2:** Factors associated with adjustment problem among first-year students at Dilla University, Dilla, southern Ethiopia, 2016(N=537).

**Characteristics**	**Adjustment problem**		** COR(95% CI)**	**AOR (95%)**
Being away from home and family	Yes	No		
No difficulty	102	254	1	1
Some difficulty	88	42	5.2(3.38,8.05)	4.15(2.46,6.99)*∗∗∗*
Great difficulty	38	12	7.8(3.96,15.70)	5.8(2.35,14.76)*∗∗∗*
Living in dormitory				
No difficulty	177	285	1	1
Some difficulty	49	20	3.9(2.3,6.9)	0.7(0.34,1.63)
Great difficulty	*2 *	*3*	1.1(0.2,6.5)	0.4(0.05,3.84)
Difficulties in Socializing or making friends				
No difficulty	*125*	*274*	1	1
Some difficulty	*94*	*30*	6.8(4.3,10.9)	3.9(2.3,6.98)*∗∗∗*
Great difficulty	*9 *	*4*	4.9(1.50,16.32)	1.1(0.25,5.21)
New, different social network & Environment				
No difficulty	* 162 *	*273*	1	1
Some difficulty	* 60 *	*31*	3.2(2.03,5.26)	0.9(0.52,1.92)
Great difficulty	* 6 *	*4*	2.5(0.70,9.09)	0.3(0.63,1.88)
Difficulties in adjusting classes				
No difficulty	*138 *	*274*	1	1
Some difficulty	*86 *	*32*	5.3(3.39,8.41)	1.5(0.7,3.32)
Great difficulty	*4 *	*2*	3.9(0.72,21.95)	0.5(0.06,5.08)
Difficulties in managing time & study skill				
No difficulty	143	*280*	1	1
Some difficulty	*75 *	*23*	6.3(3.84,10.62)	3.0(1.30,7.02)*∗∗∗*
Great difficulty	*10 *	*3*	3.9(1.31,11.67)	1.5(0.29,8.31)
Health problem				
No difficulty	*128 *	*243*	1	1
Some difficulty	*63 *	*46*	2.6(1.68,4.02)	0.9(0.5,1.61)
Great difficulty	*37 *	*49*	3.7(2.04,6.09)	0.7(0.3,1.6)

*∗∗∗*Statistically significant (P < 0.05).

## Data Availability

The data used to support the findings of this study are available from the corresponding author upon request (http://www.uog.edu.et/, wondale.getinet@uog.edu.et).

## Abbreviations

AOR: Adjusted odds ratio
COR: Crude odds ratio
OR: Odds ratio
SPSS: Statistical package for social science
SD: Standard deviation
DSM-IV: Diagnostic and statistical manual fourth
SACQ: Student Adaptation to School Questionnaire
WPS: Western Psychological Service.

## References

[1] Saleem S., Mahmood Z., N M. (2013). Mental health problems in university students: A prevalence study. *FWU Journal of Social Sciences*.

[2] Aderi M., Jdaitawi M., Ishak N. A., Jdaitawi F. (2013). The influence of demographic variables on university students' adjustment in north Jordan. *International Education Studies*.

[3] Nyamayaro PC C. S. (2013). The relationship between adjustment and negative emotional states among first-year medical students. *Asian Journal of Social Sciences and Humanities*.

[4] Association A. P. (2000). *Diagnostic and Statistical Manual-Text Revision*.

[5] Carta M. G., Balestrieri M., Murru A., Hardoy M. C. (2009). Adjustment disorder: Epidemiology, diagnosis and treatment. *Clinical Practice and Epidemiology in Mental Health*.

[6] Emmanuel O. U., TPTIB O. (2015). Adjustment Problem of National Diploma Student in Burutu Environment-A Case Study of Delta State School of Marine Technology, Burutu Delta State. *Int J of Multidisciplinary and Current research*.

[7] Shiferaw S., Fantahun M., Bekele A. (2006). Psychosocial problems among students in preparatory school, in Dessie town, north east Ethiopia. *Ethiopian Journal of Health Development*.

[8] Glenn M. C. (2011). Academic achievement and academic adjustment difficulties among college freshman. *Journal of Arts, Science, and Commerce*.

[9] Jemal J. (2011). Assessing major, adjustment problems of freshman students in Jimma University. *Ethiopian Journal of Education and Sciences*.

[10] Wang A., Chen L., Zhao B., X Y. (2006). First-Year Students' Psychological and Behavior Adaptation to College: The Role of Coping Strategies and Social Support. *Online Submission*.

[11] Yussuf A. D., Issa B. A., Ajiboye P. O., Buhari O. I. (2013). The correlates of stress, coping styles and psychiatric morbidity in the first year of medical education at a Nigerian University.. *African Journal of Psychiatry*.

[12] Gradus J. L., Qin P., Lincoln A. K., Miller M., Lawler E., Lash T. L. (2010). The association between adjustment disorder diagnosed at psychiatric treatment facilities and completed suicide. *Journal of Clinical Epidemiology*.

[13] O'Keeffe N., Ranjith G. (2007). Depression, demoralisation or adjustment disorder? Understanding emotional distress in the severely medically ill. *Clinical Medicine*.

[14] Parker J. D., Duffy J. M. (2005). Making a successful transition during the first year of college: Does emotional intelligence matter. *Invited essay January*.

[15] Gau S. S. F., Chong M. Y., Chen T. H. H., Cheng A. T. A. (2005). A 3-year panel study of mental disorders among adolescents in Taiwan. *The American Journal of Psychiatry*.

[16] Sharma B. (2012). Adjustment and emotional maturity among first year college students. *Pakistan Journal of Social and Clinical Psychology*.

[17] Van Der Klink J. J. L., Blonk R. W. B., Schene A. H., Van Dijk F. J. H. (2003). Reducing long term sickness absence by an activating intervention in adjustment disorders: A cluster randomised controlled design. *Occupational and Environmental Medicine*.

[18] Al-Qaisy L. M. (2010). Adjustment of College Freshmen: the Importance of Gender and the Place of Residence. *International Journal of Psychological Studies*.

[19] Shatkin J. P. (2007). *Transition to college: Separation and change for parents and students*.

[20] Parker J. D. A., Hogan M. J., Eastabrook J. M., Oke A., Wood L. M. (2006). Emotional intelligence and student retention: Predicting the successful transition from high school to university. *Personality and Individual Differences*.

[21] Gerdes H., Mallinckrodt B. (1994). Emotional, Social, and Academic Adjustment of College Students: A Longitudinal Study of Retention. *Journal of Counseling & Development*.

[22] Rodgers L. S., Tennison L. R. (2009). A Preliminary Assessment of Adjustment Disorder Among First-Year College Students. *Archives of Psychiatric Nursing*.

[23] Mustaffa C. S., Ilias M. (2013). Relationship between Students Adjustment Factors and Cross Cultural Adjustment: A Survey at the Northern University of Malaysia. *Intercultural communication studies*.

[24] Abdullah M. C., Elias H., Mahyuddin R., Uli J. (2009). Adjustment amongst first year students in a Malaysian university. *European Journal of Social Sciences*.

[25] Abouserie R. (1994). Sources and Levels of Stress in Relation to Locus of Control and Self Esteem in University Students. *Journal of Educational Psychology*.

[26] Magnavita N., Chiorri C. (2018). Academic stress and active learning of nursing students: A cross-sectional study. *Nurse Education Today *.

[27] Wan T.-Y., Chapman D. W., Biggs D. A. (1992). Academic stress of international students attending U.S. universities. *Research in Higher Education*.

[28] Pritchard M. E., Wilson G. S. (2003). Using Emotional and Social Factors to Predict Student Success. *Journal of College Student Development*.

